# OP10 Affordability Decision Rules: Systematic Review And Categorization Of Budget Impact Thresholds For 174 Countries Based On International Practices

**DOI:** 10.1017/S0266462324000746

**Published:** 2025-01-07

**Authors:** Andres Pichon-Riviere, Federico Cairoli, Sebastian Garcia Marti, Federico Augustovski

## Abstract

**Introduction:**

Effective health intervention coverage decision-making hinges on understanding budget impact (BI). Despite progress in estimating cost-effectiveness thresholds, a standardized approach for defining BI, particularly high BI, remains elusive. Addressing this gap, our systematic review aims to identify existing BI thresholds and establish universally applicable BI categories, providing a much-needed framework for global health policy.

**Methods:**

In our systematic review, we adhered to Cochrane methods and PRISMA reporting guidelines (PROSPERO protocol CRD42020221652). We included articles that detailed current BI or affordability thresholds used by national or regional healthcare systems, sourcing from PubMed, Embase, and International Network of Agencies for Health Technology Assessment (INAHTA) communications. To address variability across jurisdictions, we normalized BI/affordability thresholds to a fraction of each country’s total healthcare expenditure. This approach enabled us to categorize BI thresholds into four distinct levels (low, moderate, high, and very high) and apply these categories universally across countries.

**Results:**

We retrieved 1,592 records, identifying affordability thresholds and their underlying rationales in 12 countries: Argentina, Australia, England, Canada, Germany, France, Netherlands, USA, Taiwan, Ukraine, Scotland, and Singapore. Utilizing this data, we established four BI threshold levels relative to the total health budget: low (below 0.00005), moderate (0.00005 to <0.0001), high (0.0001 to <0.0002), and very high (>=0.0002). We then extrapolated these thresholds, along with their uncertainty ranges, to 174 countries, using 2022 World Bank data.

**Conclusions:**

Our study provides a comprehensive overview of current global affordability thresholds and their implications for healthcare coverage and reimbursement. We found that explicit BI thresholds are predominantly established in high-income countries. Our findings offer critical, evidence-based guidance on affordability decision rules, applicable to health systems in 174 countries, thereby contributing significantly to the standardization and informed policymaking in global healthcare.

